# Availability of services for the diagnosis and treatment of infertility in The Gambia`s public and private health facilities: a cross-sectional survey

**DOI:** 10.1186/s12913-022-08514-0

**Published:** 2022-09-07

**Authors:** Anna Afferri, Haddijatou Allen, Susan Dierickx, Mustapha Bittaye, Musa Marena, Allan Pacey, Julie Balen

**Affiliations:** 1 School of Health and Related Research – ScHARR, The University of Sheffield, Sheffield, UK; 2 Medical Research Council – MRC The Gambia Unit, Fajara, The Gambia; 3 Research Centre Gender, Diversity and Intersectionality - RHEA, Vrije Universiteit Brussel, Ixelles, Belgium; 4 The Gambia Ministry of Health, Banjul, The Gambia; 5 School of Medicine and Allied Health Sciences, University of The Gambia, Banjul, The Gambia; 6 Department of Oncology and Metabolism, The Medical School, The University of Sheffield, Sheffield, UK

**Keywords:** Infertility services, Fertility care, ART, Private care, Sub-Saharan Africa, The Gambia

## Abstract

**Background:**

Infertility is a long-standing reproductive health issue, which affects both men and women worldwide and it is especially problematic in the Global South. In sub-Saharan Africa, understanding the current availability of diagnostic and treatment services for infertility is important because this could guide health systems to improve access to fertility care for all. Yet, few studies have explicitly started from a health system perspective to grasp the availability and integration of infertility services in sub-Saharan Africa. This quantitative study, the first in The Gambia, West Africa, examines the availability of infertility services in public and private facilities as part of a wider endeavour to improve fertility care policy and practice in the country.

**Methods:**

A cross-sectional survey using Qualtrics was administered to 38 health facilities. The survey was carried out between March and August 2021 and involved closed-ended questions. Data analysis consisted of descriptive statistics and t-tests performed using SPSS version 26.

**Results:**

A total of 25 facilities (66%) offered infertility services, of which 13 (52%) were public and 12 (47%) private. Although the availability of screening tests was similar between health institutions, most diagnostic and treatment services were available only in the private sector. Treatment services included: (i) ovarian stimulation (*n* = 16, 42%); (ii) reversal of tubal ligation and/or blockage (tuboplasty) (*n* = 4, 11%); and (iii) intrauterine insemination (*n* = 3, 8%). Assisted reproductive technologies such as IVF and ICSI were not available in public or private sectors. The Gambian health management information system lacked a dedicated space to capture data on infertility. Reported barriers to integration of infertility services in existing reproductive health services included a lack of specialised training, an absence of national guidance on infertility management, and a shortage of appropriate equipment, supplies, and medication.

**Conclusions:**

The availability of infertility services in The Gambia follows a trajectory that is similar to other SSA countries in which services are mostly obtainable through the private sector. Yet, access to private care is expensive and geographically restricted, which exacerbates inequalities in accessing fertility care for all. Improving the provision of infertility services in the public sector requires systematically capturing data on infertility and investing in the provision of a full-range fertility care package.

**Supplementary Information:**

The online version contains supplementary material available at 10.1186/s12913-022-08514-0.

## Background

Infertility, a disease characterised by the failure to establish a clinical pregnancy after 12 months of regular, unprotected sexual intercourse [1]. It is an important reproductive health problem and an essential component of comprehensive sexual and reproductive health rights (SRHR) as declared at the International Conference on Population Development (ICPD), more than 25 years ago [2]. While current estimates are lacking, the most recent global survey commissioned by the WHO in 2010, indicated that up to 48 million couples suffer from infertility worldwide, with half of the global burden of infertility in low and middle-income countries (LMIC) [3–5]. Yet, this is likely to be an underestimation, as insufficient data and high fertility rates in many LMICs mask the true burden of infertility [3, 6].

The provision of infertility services in resource-poor settings is challenging [7–10]. In sub-Saharan Africa (SSA) demands for infertility services, in particular assisted reproductive technologies (ART), have increased rapidly in recent years [11–13] but despite substantial growth in demand, providing these services can be complicated and requires a range of clinical and laboratory facilities that are highly sophisticated and often very expensive [10]. This is one of the reasons why infertility services in SSA are largely confined to the private sector [7, 12, 14, 15] with few exceptions such as South Africa and Nigeria [16–18].

While several anthropological studies in SSA have investigated how the private sector navigates the delivery of infertility services [14, 19], much less research has been conducted with a national health system lens on the availability of infertility services in public facilities. There is currently limited knowledge on the management and uptake of infertility services among men and women, especially in countries where data on these services are not systematically captured and reported. Moreover, in many SSA countries, health professionals often work both the private and public sectors simultaneously which may lead to complex public–private health systems dynamics, with potential unintended consequences for patients and practitioners [20].

This study, the first of its kind in The Gambia, aims to understand the infertility services landscape in both the public and the nascent private health sector to support the inclusion of a fertility care package in the country’s sexual and reproductive health (SRH) policy and practice. This is particularly pertinent as The Gambian government has recently made strides towards the inclusion of fertility care in its national health agenda [21]. Furthermore, The Gambia is an important case study, as previous qualitative research in urban areas of the country has shown that Gambian health care providers and patients have limited knowledge of the availability of infertility services [22]. Finally, as in other SSA settings, the high fertility of Gambian women masks the true burden of infertility in the country [23] and this may diverge attention of policymakers and international donors from interventions that specifically address infertility. Studies report that there is a need for more financial and logistic support, and there is a shortage of adequately and appropriately trained health staff involved in fertility care provision [21, 24].

This study, which builds on previous work in The Gambia and is part of wider body of work, presents the results of a country-wide quantitative cross-sectional survey including public and private health facilities and assess the availability and distribution of infertility services.

## Methods

### Study setting

The Gambia is a West African country that shares a border with Senegal. The country has an estimated population of 2.3 million inhabitants and a markedly diverse profile, with approximately 60% of the population living in coastal urban areas and the remaining 40% in rural areas [23]. As a result of economic instability and colonial and postcolonial politics, the national health system faces many challenges [25]. Changes in the political environment since 2016 have helped encourage the emergence of private health providers, both *for profit* and *not-for-profit* [26].

The Gambian public health system has a decentralised structure, distributed across three tiers, namely primary, secondary and tertiary levels [25]. The public sector is represented by Edward Francis Small Teaching Hospital (the main referral hospital of the Gambian health system), district (*n* = 4) and general hospitals (*n* = 5), major and minor health centres and a plethora of rural health posts and village-based health services. The rapidly expanding private health sector is composed of clinics and medical centres, mainly concentrated in urban areas in the Western regions [27]. Research on infertility was conducted in The Gambia over two decades ago [28, 29], and has recently resumed [30–35]. Anyanwu and Idoko (2017) estimated the prevalence of infertility in The Gambia at 14.3%, allocating its etiology to female (tubal) secondary infertility and male (sperm) factors [35].

### Study design

This is a cross-sectional study conducted via a survey questionnaire administered in person to public facilities and private clinics across The Gambia. Thirty-eight (*n* = 38) health facilities participated in the survey including 20 (53%) public and 18 (47%) private. Private clinics operated *for-profit* (*n* = 8) and *not-for-profit* (*n* = 10), whereby *not-for-profit* means that the facility is supported financially by a non-governmental organisation or charity. For the aim of this study, the private clinics were disaggregated into these two arms to investigate any possible difference in the provision of infertility services based on the profit status.

### Sample size and recruitment

The sample of health facilities was extrapolated from an exhaustive list provided by the Gambian Bureau of Statistics and via multiple interactions with Gambian health experts, and included both public facilities and private clinics. For the purpose of this study, only health facilities representing secondary and tertiary levels of care were selected. Primary-level facilities (village health posts) were not included in the sample because they do not offer any infertility services, but only offer referrals to the upper levels of care. The sample of public facilities included major health centres, district and general hospitals, and the teaching hospital [26]. These public facilities were recruited in-toto, and represented the entirety of the facilities in these levels of care.

However, during the data collection, it was discovered that some public facilities labelled as major centers were in fact minor centers (*n* = 6). Because the sampling and recruitment had already taken place, they were kept in the study for completion purposes.

Given the small sample size, private clinics were selected from the list obtained by the government and simply randomised to have an equal chance of selection for inclusion in the sample. For random selection, we used an online tool that generated a random order of private clinics. Random selection eliminates selective biases and is the only effective strategy for obtaining representative samples. Due to the lack of an updated census for the private sector, during the data collection we came across two additional private clinics. These two additional clinics belonged to the same population as the original sample, with a similar time frame for the data collection and they were therefore included in the sampling.

Prior to the study implementation, permission was requested from the Ministry of Health (MoH), and the purpose of the study was discussed with the seven Regional Health Directorates. All facilities were contacted, and their participation was requested using an official invitation letter with information about the study and details of the ethical approval (see below). An eligible respondent per facility was nominated and then contacted directly by the trained Gambian researcher (HA) and invited to take part in the study. The respondents included health facilities staff, such as medical doctors, gynaecologists, nurses, and midwives who provided information on available infertility services. These key informants were invited to participate because they have relevant knowledge or expertise in fertility care within their organisational settings.

### Quality control

The survey questionnaire was pre-tested in two sites in the urban area of Kanifing—Western Region (one public facility and one private clinic). No major modifications were introduced to the tool after testing. Due to the small original sample size, data from the piloted facilities were included in the final analysis.

### Data collection

A computer-assisted personal survey was conducted by either the Gambian Research (HA) or the study lead (AA) in the offices, clinic rooms and wards of each facility. The questionnaire was written and administered in English, the official and working language of The Gambia. Data collection was conducted from March to August 2021.

The survey questionnaire contained 36 closed-ended questions which required respondents to provide information on various aspects of fertility care provision. The questions were categorised into six sections and included: (i) demographic information on the professional qualification and gender of the respondents; (ii) characteristics of the study sites (name and location of the facility, level of care); (iii) the availability of reproductive health services including infertility services and personnel; and (iv) the health management system. The remaining two sections included two 4-point Likert scales [36] to help better understand the relevance of key barriers to integration of infertility services. The survey was developed using the web-based Qualtrics XM software version 10, 2021©. An additional file shows this in more detail (see Additional file 1).

### Data analysis

Data were analysed using IBM Statistical Package for the Social Sciences (SPSS) for Windows, version 26.0. The primary analysis applied descriptive statistics using frequencies and cross-tabulation for the main outcome variables. Likert scales were used to rank barriers to integration of infertility services into existing SRH services. Statistical significance was established at *p* < 0.05. Fisher’s exact test was used for analysis of contingency tables.

### Ethical approval

Ethical approval was obtained from The Gambia Government and Medical Research Council (MRCG) at the London School of Hygiene and Tropical Medicine Joint Ethics Committee (Reference 22,446) and the University of Sheffield – School of Health and Related Research (ScHARR) Research Ethics Committee (Reference 03,785–038,109). Written informed consent was obtained from all respondents prior to the beginning of the data collection.

## Results

### Characteristics of participating institutions and survey respondents

**Fig. 1:**
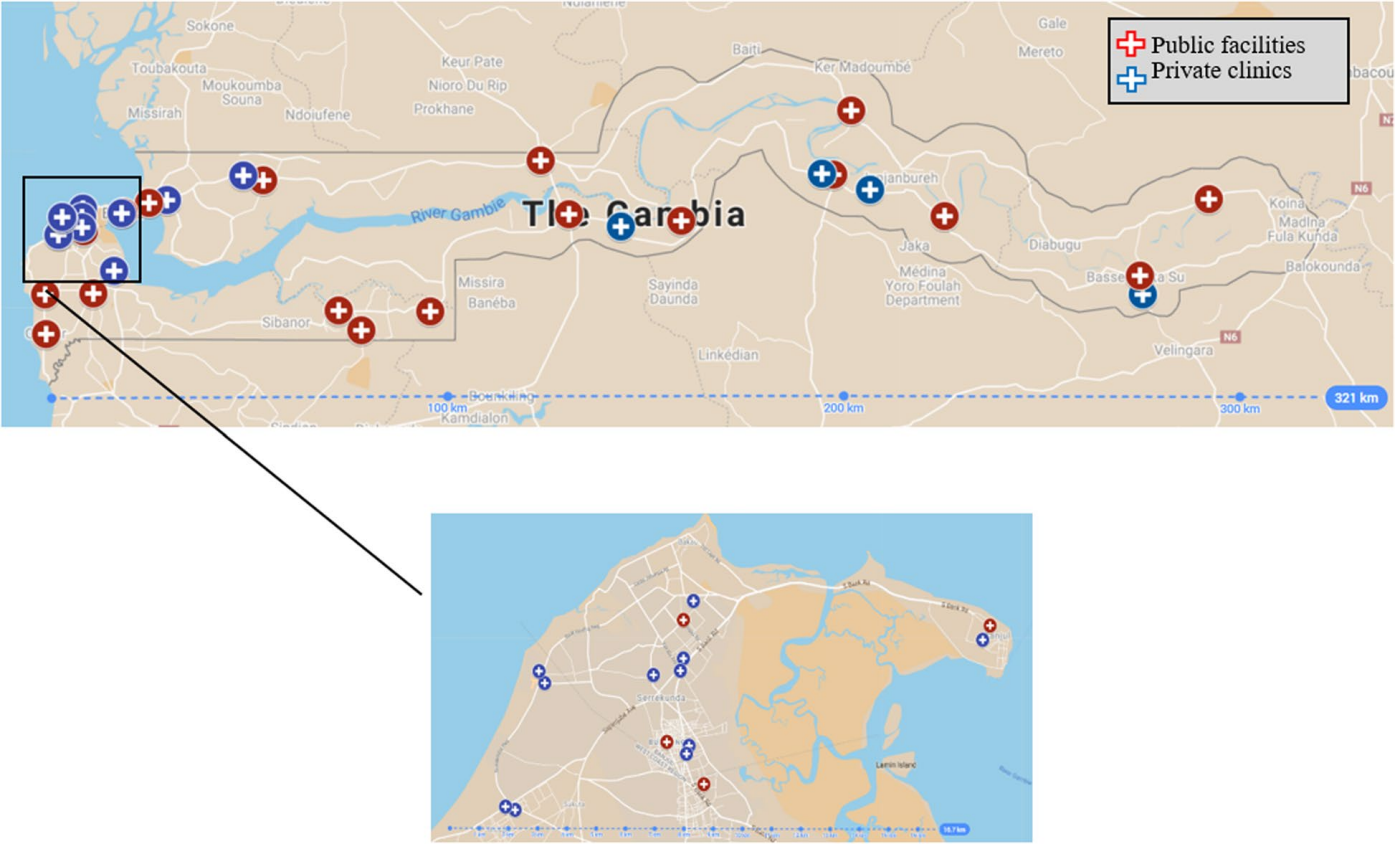
GIS location of the health facilities surveyed with the Greater Banjul Area magnified

Slightly over half of the participating institutions (*n* = 22; 58%), including the teaching hospital, were located in the Western regions 1 and 2, including the Greater Banjul Area. The remaining sixteen (42%) were distributed throughout the country (Fig. 1).

A total of 45% (*n* = 17) of the survey respondents were medical doctors and 55% (*n* = 21) were nursing or midwifery staff; the vast majority (*n* = 30; 79%) were male (Table 1).

**Table 1:** Key characteristics of the participating respondents and facilities, and overall (*n* = 38)

	***Public facilities*** ***(n* = *20; 53%)***	***Private clinics*** ***(n* = *18; 47%)***	***TOTAL*** ***(n* = *38; 100%)***
**Respondent role**			
*Doctor (gynaecologist)*	*2 (10%)*	*6 (33%)*	***8 (21%)***
*Doctor (physician, physician assistant, medical officer, medical assistant)*	*7 (35%)*	*2 (11%)*	***9 (24%)***
*Nurses-Midwives*	*11 (55%)*	*10 (56%)*	***21 (55%)***
**Respondent gender**			
*Male*	*15 (75%)*	*15 (83%)*	***30 (79%)***
*Female*	*5 (25%)*	*3 (17%)*	***8 (21%)***
**Level of care**			
*Secondary*	*10 (50%)*	*13 (72%)*	***23 (61%)***
*Tertiary*	*10 (50%)*	*5 (28%)*	***15 (39%)***
**Type of private clinics**			
*For-profit*		*7 (39%)*	***7 (39%)***
*Not-for-profit*		*11 (61%)*	***11 (61%)***

### Provision of infertility services in public facilities and private clinics

Infertility services were provided in 25 (66%) of the surveyed facilities, specifically in 13 out of 20 (65%) public facilities and 12 out of 18 (67%) private clinics with no statistical difference in the overall infertility service availability between the two sectors (*p* = 0.06). Most (16/25; 64%) facilities that provided infertility services, were located in the Western regions, specifically in the Greater Banjul Area that include the Senegambia, Brusubi, Kanifing and Brikama districts. Furthermore, among these 25 facilities, four (11%) were classified as minor health centres, six (16%) as major health centres, five (13%) as district hospitals, nine (24%) as general hospitals, and one (3%) as a teaching hospital, located in the capital Banjul and representing the referral point for the entire Gambian health system (Table 2).

**Table 2:** Infertility services by level of care among the surveyed public facilities and private clinics, and overall (*n* = 38)

	***Public facilities*** ***(n* = *20; 53%)***	***Private clinics*** ***(n* = *18; 47%)***	***TOTAL*** ***(n* = *38; 100%)***
**No infertility services**	**7 (35%)**	**6 (33%)**	**13 (34%)**
**Infertility services**	**13 (65%)**	**12 (67%)**	**25 (66%)**
**Secondary level**			
*Minor health centres*	*1 (5%)*	*3 (17%)*	***4 (11%)***
*Major health centres*	*2 (10%)*	*4 (22%)*	***6 (16%)***
**Tertiary level**			
*District hospitals*	*4 (20%)*	*1 (6%)*	***5 (13%)***
*General hospitals*	*5 (25%)*	*4 (22%)*	***9 (24%)***
*Teaching hospital*	*1 (5%)*	*0*	***1 (3%)***

### Infertility screening and diagnostic services in public facilities and private clinics

Most of the institutions that offered infertility services were able to collect patient medical history and perform physical examinations for men and women. Compared to private clinics, public facilities had a slightly increased capacity to undertake testing for Human Immunodeficiency Virus (HIV) and Tuberculosis (TB). Sexually transmitted infections (STI) tests were equally available in both the public facilities and private clinics. However, the ability to carry out key diagnostic components of infertility services was generally higher in the private sector. Specifically, female hormonal profiling was not available in public facilities, and tubal patency investigations such as hysterosalpingogram (HSG) and sonohysterosalpingogram (SHG) were available in only one out of 20 (5%) public facilities.

Finally, pelvic ultrasound and semen analysis were available in 47% (*n* = 18) and 42% (*n* = 16) of the facilities, respectively (Table 3).

**Table 3:** Details of infertility screening and diagnostic services in public facilities and private clinics, and overall (*n* = 38)

	***Public facilities*** ***(n* = *20; 53%)***	***Private clinics*** ***(n* = *18; 47%)***	***TOTAL (n* = *38; 100%)***
**No screening and diagnostic services**	**7 (35%)**	**6 (33%)**	**13 (34%)**
**Screening and/or diagnostic services**^a^	**13 (65%)**	**12 (67%)**	**25 (66%)**
**Screening/diagnostic (general)**			
*Fertility history-taking*	*12 (60%)*	*12 (67%)*	***24 (63%)***
*Physical examination (female)*	*12 (60%)*	*11 (61%)*	***23 (61%)***
*Physical examination (male)*	*11 (55%)*	*11 (61%)*	***22 (58%)***
**Screening (female)**			
*STIs*	*12 (60%)*	*12 (67%)*	***24 (63%)***
*HIV*	*12 (60%)*	*10 (56%)*	***22 (58%)***
*TB*	*12 (60%)*	*6 (33%)*	***18 (47%)***
*Visual inspection w/ acetic acid*	*7 (35%)*	*5 (28%)*	***12 (32%)***
*Smear test*	*5 (25%)*	*5 (28%)*	***10 (26%)***
**Screening (male)**			
*STIs*	*11 (55%)*	*11 (61%)*	***22 (58%)***
*HIV*	*12 (60%)*	*9 (50%)*	***21 (55%)***
*TB*	*11 (55%)*	*6 (33%)*	***17 (45%)***
**Diagnostic testing (female)**			
*Ultrasound (pelvic)*	*8 (40%)*	*10 (56%)*	***18 (47%)***
*Hormonal profile*	*0 (0%)*	*8 (44%)*	***8 (21%)***
*Hysterosalpingogram (HSG)*	*1 (5%)*	*7 (39%)*	***8 (21%)***
*Sonohysterosalpingogram (SHG)*	*1 (5%)*	*3 (17%)*	***4 (11%)***
**Diagnostic testing (male)**			
*Semen analysis*	*7 (35%)*	*9 (50%)*	***16 (42%)***

^a^ Clinics can offer more than one type of services

When examining the difference between the private sector *for-profit* and *not-for-profit*, more screening and diagnostic services were available in the former with semen analysis, female hormonal profile, HSG and SHG mostly available in the *for-profit* sector (Table 4).

**Table 4:** Details of infertility screening and diagnostic services in the private sector for profit and not-for-profit, and overall (*n* = 18)

	***For-profit*** ***(n* = *8; 44%)***	***Not-for-profit*** ***(n* = *10; 55%)***	***TOTAL*** ***(n* = *18; 100%)***
**No screening or diagnostic services**	**0**	**6 (60%)**	**6 (33%)**
**Screening and diagnostic services**^**a**^	**8 (100%)**	**4 (40%)**	**12 (67%)**
*STIs (both female and male)*	*8 (100%)*	*4 (40%)*	***12 (67%)***
*Ultrasound (pelvic)*	*7 (88%)*	*3 (30%)*	***10 (56%)***
*Semen analysis*	*8 (100%)*	*1 (10%)*	***9 (50%)***
*Hormonal profile (female)*	*7 (88%)*	*1 (10%)*	***8 (44%)***
*Hysterosalpingogram (HSG)*	*7 (88%)*	*0*	***7 (39%)***
*Sonohysterosalpingogram (SHG)*	*3 (38%)*	*0*	***3 (17%)***

^a^ Clinics can offer more than one type of services

### Infertility treatment services in public facilities and private clinics

Regarding infertility treatments, 16 facilities (42%) provided dilation and curettage (D&C), 16 (42%) offered ovulation induction with Clomiphene citrate or Letrozole, and six (16%) performed varicocele repair surgery. Finally, four facilities (11%) were able to perform reversal of tubal ligation through tuboplasty (one public facility and three private clinics) (Table 5). Three (8%) facilities all of which were private and located in the Western regions, offered Intrauterine Insemination (IUI) and only one (3%) was able to perform vasectomy reversal. ART such as *in-vitro* fertilisation (IVF) and intracytoplasmic sperm injection (ICSI) were reported as not available in The Gambia at the time of data collection.

**Table 5:** Details of infertility treatment services in public facilities and private clinics, and overall (*n* = 38)

	***Public facilities*** ***(n* = *20; 53%)***	***Private clinics*** ***(n* = *18; 47%)***	***TOTAL*** ***(n* = *38; 100%)***
*Dilation and curettage*	*8 (40%)*	*8 (44%)*	***16 (42%)***
*Ovulation induction*	*7 (35%)*	*9 (50%)*	***16 (42%)***
*Varicocele repair*	*3 (15%)*	*3 (17%)*	***6 (16%)***
*Reversal of tubal ligation/blockage*	*1 (5%)*	*3 (17%)*	***4 (11%)***
*Intrauterine insemination (IUI)*	*0*	*3 (17%)*	***3 (8%)***
*Reversal of vasectomy*	*0*	*1 (6%)*	***1 (3%)***
*In-vitro fertilisation (IVF)*	*0*	*0*	***0***
*Intracytoplasmic sperm injection (ICSI)*	*0*	*0*	***0***

### Infertility service delivery and monitoring

More than half of the facilities (*n* = 26/38; 68%) reported that they consulted between 0–25 clients per week for infertility, but the time taken for infertility consultations was said to absorb a limited amount of time and only slightly increased the workload of the health providers. To this effect, 58% (*n* = 22) of the respondents reported they spent between 0 and 25% of their time consulting for infertility. No statistical difference was observed between sectors (*p* = 0.38).

Approximately half of the facilities (17/38, 45%) reported that 51%-75% of the infertility consultations were attended by women; 8 facilities (21%) reported that in 75%-99% the consultations for infertility are attended by women and finally, 4 facilities (11%) cited that 100% of their consultation are attended by women. In just under half (*n* = 18; 47%) of the initial infertility consultation, the partners never attend together but this altered in subsequent visits with a cumulative 58% (*n* = 22) of respondents reporting that ‘often’ and ‘usually’ one partner accompany the other partner during a follow-up visit.

Twenty-three out of 38 facilities (61%) indicated that they do not report any data on infertility to the MoH via the Health Management Information System (HMIS) or by any other means. Specifically, 10 (50%) public facilities and 13 (72%) private clinics did not capture infertility data. In a few cases (*n* = 10; 26%) data on infertility was cited as captured and reported to the MoH using the current HMIS form. (Table 6). However, was not clear how the facilities collect and report this data.

**Table 6:** Delivery and monitoring of infertility services in public facilities and private clinics, and overall (*n* = 38)

	***Public facilities*** ***(n* = *20; 53%)***	***Private clinics*** ***(n* = *18; 47%)***	***TOTAL (n* = *38; 100%)***
**Attending initial visit as couple**			
*Partner never present*	*10 (50%)*	*8 (44%)*	***18 (47%)***
*Partner often/usually present* *Partner occasionally present*	*2 (10%)* *8 (40%)*	*3 (16%)* *7 (39%)*	***5 (13%)*** ***15 (39%)***
**Attending follow-up visits as couple**			
*Partner never present*	*5 (25%)*	*2 (11%)*	***7 (18%)***
*Partner often/usually present* *Partner occasionally present*	*9 (45%)* *6 (30%)*	*13 (72%)* *3 (17%)*	***22 (58%)*** ***9 (24%)***
**Capturing and reporting data on infertility**			
*Yes*	*7 (35%)*	*3 (16%)*	***10 (26%)***
*No*	*10 (50%)*	*13 (72%)*	***23 (61%)***
*Do not know*	*3 (15%)*	*2 (11%)*	***5 (13%)***

### Integration of infertility services into existing sexual and reproductive health services

**Fig. 2:**
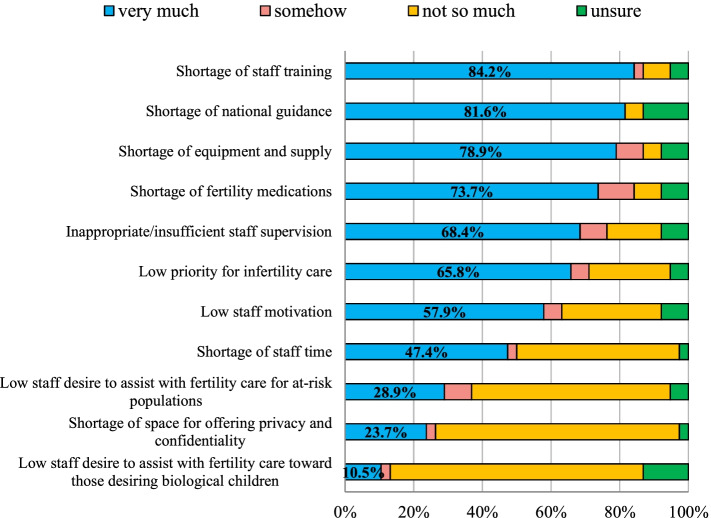
Reported barriers for integration of infertility services into existing sexual and reproductive health services in The Gambia

Of the 25 facilities that offered infertility services, just over half (*n* = 13; 52%) offered them five-days a week. In 22 (88%) of the 25 facilities, infertility services were integrated into existing reproductive health services, mainly within gynaecology, family planning or maternal health clinics. However, three *for-profit* clinics provide a standalone service dedicated solely to fertility care patients. Overall, most (*n* = 32; 84%) respondents felt that a lack of specialised training was the strongest impediment to full integration of infertility services in their facility, followed by the absence of national guidance on infertility management (*n* = 31; 82%) and a shortage of appropriate equipment, supplies and medications, respectively (*n* = 30, 79%; *n* = 28, 74%). Low policy priority for infertility was cited as the sixth main barrier to integration (*n* = 25; 66%) (Fig. 2).

## Discussion

The provision of infertility services in The Gambia is characterised by major inequalities, including uneven distribution among urban and rural settings, fragmentation across health sectors, and large geographical distances between communities and clinics. Basic investigations for infertility are generally available in public health facilities. These include medical history-taking, physical examination for both women and men, and screening for STIs, HIV and TB as aligned with national disease control programmes [37]. Although public and private facilities show comparable screening capacity, this trend reverses once diagnostic and treatment services are taken into consideration. In this sense, the private sector provides an increasing proportion of diagnostic and treatment services. For example, intrauterine insemination, a relatively simple and low cost fertility treatment that is considered the first-line treatment for mild male-factor and unexplained infertility [38–40], is unavailable in public facilities. These differences are even more accentuated between *for-profit* and *not-for-profit* clinics, where more sophisticated treatments are available in the former.

This picture is not surprising and aligns with market opportunities currently emerging in The Gambia that appear to reflect a trend seen in many countries across SSA [10, 14]. Private clinics exclusively offering fertility care have begun to emerge in recent years, indicating a growing trend for the future provision of infertility services in the country, and across the continent [7]. In The Gambia, while many of the surveyed public facilities offered some type of infertility services, the leading role of the private sector—in particular for infertility treatments – is in sharp contrast with what is offered in the public sector. Although, high costs of private fertility care are likely to also increase inequalities, raising questions about reproductive justice in the country [22, 38, 41, 42]. We assume that the lack of a formal national fertility care package and/or infertility management guidelines contributes to this unequal availability of infertility services between the two sectors, and this may potentially encourage the private sector to develop its own standards. Having established procedures for infertility management is therefore an essential step for the Gambian health system, in order to provide safe and effective high-quality infertility care.

Also, a more robust collaboration and partnership with the private sector may fill the gaps in the provision of services in the public sector and increase affordability via, for example, subsidisation of care and compliance with the Universal Health Coverage and reproductive health rights fundamentals [43].

Recently, The Gambia has enacted the National Health Insurance Scheme (NHIS) bill, which will cover the cost of an essential health care package for all who register, at a much-subsidised cost. The envelope of diseases and conditions covered by the insurance will be reviewed periodically and expanded as it becomes more affordable. In this regard, this study may help Gambian health policy and decision-makers to initiate discussions to improve infertility diagnosis and treatment, and to implement accessible and affordable infertility services for those in need. Although infertility is not directly linked to an increased mortality rate, research in The Gambia [30, 31] and elsewhere in Africa [44–46] clearly illustrates the significant social and economic burden of infertility and its impact on gender equity, suggesting that this condition should no longer be ignored [47].

Given that the majority of institutions reported most of the consultation were attended by women and the members of the couple visit the facility alone, we can conclude that women attend initial consultations for infertility without their partner [48]. Although in approximately half of initial consultations by women for fertility problems their partner was not present (results are comparable in both health sectors), the preference of the medical practitioners is to manage infertility as a *couple’s issue* [34]. This is further confirmed in this study, which indicate that male partners are more involved in infertility treatment in follow-up visits compared to initial visits. These findings also illustrate similarity with a studies conducted in SSA [34, 49, 50]. Surprisingly, in our study, less than 25% of the consultations are reportedly for male factor infertility, as many studies [51–54] conducted elsewhere have found that the causes of infertility are equally split between genders (in heterosexual couples). This could also be the result of men attending visits of their spouses without being themselves diagnosed. Research has previously shown that stigma surrounding fertility problems for men could results in poor health-seeking behaviour, and increase the already scarce male involvement in the therapeutic journey [34]. It is imperative to understand the aetiology of infertility issues from a Gambian perspective. In fact, as our work indicates, male infertility services are still poorly accessed, and mainly limited to investigation of semen parameters. Treatment for men is essentially restricted to varicocele surgery [55] although there is relatively little evidence that fertility is increased after such surgery [56]. The availability of male hormone testing was not assessed during the survey, but considering the paucity of facilities that offer female hormone profiling, it can be assumed that male hormone testing is also limited in The Gambia [57].

This study found that almost half of the surveyed facilities (42%) offered D&C. It was not clear, however, whether this procedure is linked to the provision of infertility treatment or if it is just one of the services offered to treat gynaecological issues. Previous research although outdated, suggests that this practice might be relatively common in The Gambia [29]. Further investigation is required to understand if this practice is endorsed by the Gambian medical institutions as one of the potential treatment for infertility. Moreover, it is important to note that the literature is discordant in supporting or contradicting endometrial injury as a preparatory step before medically assisted reproduction [58–60]. Given that The Gambia does not currently offer any ART however, D&C is likely to be limited to the treatment of specific gynaecological conditions [61]. As noted above, guidelines on infertility management should be developed and aligned with international evidence-based standards.

The Gambian HMIS lacks a dedicated space to capture data on infertility. Particularly, the current data collection form does not appear to systematically capture data on infertility, making it difficult to estimate the true demand for and access to infertility services in the country. Obtaining reliable national estimates of infertility services is critical and might increase the attention of policy makers and international donors [3, 62]. In this regard, The Gambian health system may consider adapting its HMIS form to collect consultations for infertility-related issues, disaggregated by sex, in a systematic and comprehensive way. This could stimulate The Gambia to investigate further concerning the true prevalence of infertility in its population and to adapt its reproductive health services for greater inclusion of neglected issues, especially among men [34].

Finally, most of the participants cited a lack of specialised infertility training, an absence of national guidance on infertility management, and a shortage of investment in appropriate equipment, supplies and medication as key barriers to full integration of infertility services into existing reproductive health services. These findings corroborated those from a recent qualitative evidence synthesis conducted in African settings [63] and with studies in other LMIC [64] and highlight, once more, the need to implement a full range of fertility care interventions regulated by national and international policy guidelines [48].

It is important to highlight the study limitations. First, not having an updated census of private clinics might have masked those recently established but not yet listed under the MoH. In this regard, the two additional private clinics included in the study sample were discovered coincidentally, and were established in the six months preceding the survey. Secondly, six of the public facilities that had been labelled as major health centers were indeed minor centers, having been downgraded prior to the study commencement. Third, different sources in The Gambia shown dissimilar figures for the number of health facilities currently functioning, and figures from the national HMIS are not always consistent with those provided by the MoH. We selected to use the latter given that these were more readily available to the study team. Lastly, we did not conduct any direct observations or patient interviews as these were out of the scope of this study. Future work may wish to explore the clinical experiences of patients and providers to better understand the provision of infertility services in the Gambian health system.

## Conclusions

The availability of infertility services in The Gambia follows a trajectory that is similar to other SSA countries in which services are limited and obtainable mostly through the private sector. In The Gambia infertility services are limited and unequally split between public and private sectors and this picture is even more distinct for the provision of infertility treatment. Furthermore, access to private care is expensive and geographically restricted, likely exacerbating existing inequalities to fertility care. Improving the availability of infertility services in the public sector requires systematically capturing data on infertility and investing in fertility training, medications, and equipment.

The Gambia Government, with the recent revision of its national health policy, laid the foundations to increase the availability of infertility services to its citizens. This may also be an opportunity to partner with the private healthcare sector as a possible option to limit the financial burden of out-of-pockets expenses on infertility services in particular among those most in need [65].

## Supplementary Information

**Additional file 1.** Survey questionnaire.

## Data Availability

The datasets generated and analysed during the current study are not publicly available because they contain personal information regarding the participants but are available from the corresponding author upon reasonable request.
