# Supplementary material for: Availability of services for the diagnosis and treatment of infertility in The Gambia`s public and private health facilities: a cross-sectional survey
Source: BMC Health Serv Res. 2022 Sep 7;22:1127. doi: 10.1186/s12913-022-08514-0 (PMC9450453; doi:10.1186/s12913-022-08514-0)
Supplement: Supplementary file 1 — Additional file 1. Survey questionnaire. [file 12913_2022_8514_MOESM1_ESM.docx]

**INFERTILITY SERVICES**

1. **Consent:**

*Now that you have read the participant information sheet and given written consent, do you have any questions about the survey?*

*Do I have your agreement to proceed with the questions?*

1. YES, I consent
2. No, I do not consent (End the survey)
3. **Demographics:**

Local Governmental Area: (window menu)

District: (window menu)

Health facility name: __________________

Level of care:

1. Primary
2. Secondary
3. Tertiary

Facility type:

1. Minor health center
2. Major health center
3. District hospital
4. General Hospital
5. Specialised hospital
6. Teaching hospital

Is this facility

1. Public
2. Private (for-profit)
3. Private (not-for-profit)
4. Other:________________________

Facility catchment population: ___________

Gender of the interviewee:

1. Male
2. Female
3. Prefer not to say

GIS points:

Longitude:____________________

Latitude:______________________

1. **SRH Services**
2. Which of the following sexual and reproductive health services are offered in this facility? (Read all the options below and select all that apply)

|  | **YES/NO** |
| --- | --- |
| Family planning |  |
| STIs management |  |
| Maternal, newborn and child care |  |
| HIV/AIDS |  |
| Prevention and management of gender-based violence (SGBV) |  |
| Prevention of unsafe abortion and post-abortion care |  |
| Andrology |  |
| Urology |  |
| Gynaecology |  |
| Obstetrics |  |
| Adolescents sexual and reproductive health |  |
| Early pregnancy/recurrent pregnancy loss clinic |  |
| Genetic screening |  |
| NONE OF THEM |  |

1. Do you offer counselling regarding optimising natural fertility (lifestyle factors, prenatal nutrition and vitamins, timing of intercourse – and other ovulation detection methods)?
   1. YES
   2. NO
   3. Do not know
2. Do you offer infertility services?
   1. YES (*skip to question 4*)
   2. NO (*skip to question 9*)
   3. Do not know (*skip to question 9*)
3. Which infertility services do you offer? (Read all the options below and check the case if the service is mentioned)

|  | **YES/NO** |
| --- | --- |
| Fertility history-taking |  |
| Physical examination (female) |  |
| Physical examination (male) |  |
| *Screening (female)* |  |
| - STIs |  |
| - HIV |  |
| - TB |  |
| - Pap test/smear test |  |
| - Visual inspection with Acetic Acid |  |
| *Screening (male)* |  |
| - STIs |  |
| - HIV |  |
| - TB |  |
| *Diagnostic testing (female)* |  |
| - Ultrasound (pelvic) |  |
| - Hormones |  |
| - Sono-hysterosalpingogram (SHG) |  |
| - Hysterosalpingogram (HSG) |  |
| *Diagnostic testing (male)* |  |
| - Semen analysis |  |

1. Do you offer any of the following female infertility treatments? (Read all the options below and select all that apply)
2. Intrauterine insemination - IUI
3. Ovulation induction (gonadotropins & hormones)
4. Reversal tubal sterilisation
5. Dilatation & Curettage/ Endometrial scratching
6. No treatment offered
7. Other treatment or surgeries for female infertility?
   1. Yes, which ones?: ___________________
   2. No
8. Do you offer any of the following male infertility treatments? (Read all the options below and select all that apply)
   1. Reversal vasectomy
   2. Surgical sperm retrieval
   3. Varicocele repair
   4. None of them
9. Which of the following Assisted Reproduction Technologies (ART) do you offer in this facility? (Read all the options below and select all that apply)
   1. In-vitro Fertilization (IVF)
   2. IVF with Intracytoplasmic Sperm Injection (ICSI)
   3. Embryo cryopreservation
   4. Oocyte cryopreservation
   5. Sperm cryopreservation
   6. Pre-implantation genetic diagnosis/screening (PGD/PGS)
   7. Sex selection
   8. None of them
10. Who provides infertility services in this facility? (Read all the options below and select all that apply)

| Physicians |
| --- |
| Obstetrician/Gynaecologists |
| Endocrinologists |
| Anaesthetists |
| Medical assistants |
| Pharmacists & pharmacist assistants |
| Nurses |
| Midwives |
| Embryologists |
| Laboratory scientists & assistants |
| Andrologists |
| Psychologists or Counsellors |
| Do not know |

1. How frequently are infertility consultations offered? (Read all the options below and select all that apply)
   1. Daily (Mon-Fri)
   2. Weekly (once a week)
   3. Monthly
   4. On request
   5. Do not know
2. Are infertility consultations stand-alone, or integrated with other services? (Integrated = health service is organised so that people get the care they need when they need it; Stand-alone = vertical service provided separately from the health facility)
   1. Stand-alone (*skip to question 12*)
   2. Integrated

If INTEGRATED, within what service?

1. Gynaecology clinic
2. Family Planning clinic
3. Maternal health clinic
4. HIV clinic
5. Other, please specify________________
6. How many patients or clients per week, are seen at this clinic for infertility?
   1. Zero
   2. Fewer than 5
   3. Between 6 and 25
   4. Between 26 and 50
   5. Between 51 and 70
   6. More than 70
   7. Do not know
7. What is the percentage of the total time spent, by the health staff, providing infertility-related consultations at this clinic?
8. 0-25%
9. 26-50%
10. 51-75%
11. 76-99%
12. 100%
13. Do not know
14. What percentage of all infertility consultations address female fertility issues?
15. 0%
16. 1% - 25%
17. 26% - 50%
18. 51% - 75%
19. 76% - 99%
20. 100%
21. Do not know

1. What percentage of all infertility consultations address male infertility issues?
2. 0%
3. 1% - 25%
4. 26% - 50%
5. 51% - 75%
6. 76% - 99%
7. 100%
8. Do not know
9. During the first visit for infertility, do couples attend together? (Read all the options below and select all that apply)
   1. Always
   2. Usually
   3. Often
   4. Occasionally
   5. Never
10. During follow-up visits for infertility, do couples attend together? (Read all the options below and select all that apply)
11. Always
12. Usually
13. Often
14. Occasionally
15. Never
16. Do you report data about infertility to the District Health Management Information System?
17. Yes
18. No (*skip to question 20*)

If YES, how do you report data concerning infertility? (Please take a picture of the form, if available)

- 1. DHMS paper form
  2. DHMS electronic form
  3. Data is added to the total number of outpatient consultations
  4. Other formats (please specify)_____________________

1. How often do you report data concerning infertility consultations?
2. Weekly
3. Monthly
4. Bi-annually
5. On demand
6. This data is not reported
7. Does this health facility follow any specific ethical protocols when it comes to providing infertility treatment?
   1. Yes
   2. No
   3. Don’t know
   4. Not applicable, the health facility does not provide infertility treatment

If YES, could you explain which ethical protocols are being followed? (Please take a picture of the protocol, if available)

- 1. Checklist or protocol developed by this health facility
  2. National ethical guidelines regarding the provision of infertility services
  3. Other, please specify___________________

1. Does this health facility have an ethical board meeting to discuss ethically sensitive requests regarding infertility treatment?
   1. Yes
   2. No
   3. Don’t know
2. Who commonly makes the final decision when it comes to ethically sensitive request for infertility treatment?
   1. The woman wanting infertility treatment
   2. The man wanting infertility treatment
   3. Decision are always made together by both man and woman wanting infertility treatment
   4. The medical doctor providing treatment
   5. The ethical board of this health facility
   6. Other, specify ______________________
3. Could you say, for each factor, how much each barrier impede integration of infertility care? (Read all the options below and select all that apply)

|  | 1  (not so much) | 2 (somehow) | 3  (very much) | 4  Unsure |
| --- | --- | --- | --- | --- |
| Shortage of staff time | ⃝ | ⃝ | ⃝ | ⃝ |
| Shortage of staff training | ⃝ | ⃝ | ⃝ | ⃝ |
| Inappropriate/insufficient staff supervision | ⃝ | ⃝ | ⃝ | ⃝ |
| Low staff motivation | ⃝ | ⃝ | ⃝ | ⃝ |
| Low staff desire to assist with fertility care due to stigmatization toward those desiring biological children | ⃝ | ⃝ | ⃝ | ⃝ |
| Low staff desire to assist with fertility care for at-risk populations (such as those with HIV, disabled people, etc.) | ⃝ | ⃝ | ⃝ | ⃝ |
| Shortage of fertility medications | ⃝ | ⃝ | ⃝ | ⃝ |
| Shortage of equipment and supply | ⃝ | ⃝ | ⃝ | ⃝ |
| Shortage of space for offering privacy and confidentiality | ⃝ | ⃝ | ⃝ | ⃝ |
| Shortage of national guidance | ⃝ | ⃝ | ⃝ | ⃝ |
| Low priority for infertility care | ⃝ | ⃝ | ⃝ | ⃝ |

1. In the case infertility care is integrated with the existing services in your facility, would this lead to an increase or decrease of the following components? (Read all the options below and select all that apply)

|  | Decrease | No change | Increase | Unsure |
| --- | --- | --- | --- | --- |
| Cost of services (for the facility) | ⃝ | ⃝ | ⃝ | ⃝ |
| Cost of services (for the client) | ⃝ | ⃝ | ⃝ | ⃝ |
| Efficiency of services | ⃝ | ⃝ | ⃝ | ⃝ |
| Workload for the staff | ⃝ | ⃝ | ⃝ | ⃝ |
| Time spent per client | ⃝ | ⃝ | ⃝ | ⃝ |
| Equipment, supplies, and drugs for infertility treatment | ⃝ | ⃝ | ⃝ | ⃝ |

1. **Follow-up**

Do you agree to be contacted for a qualitative interview in about 6 months’ time?

- 1. Yes

Contact name: ___________________

Occupation: _____________________

Contact number: __________________

- 1. No

If NO, can you name a person, from this health facility, that may agree to be interviewed?_________________________
